# Remote Assessment of Disease and Relapse in Epilepsy: Protocol for a Multicenter Prospective Cohort Study

**DOI:** 10.2196/21840

**Published:** 2020-12-16

**Authors:** Elisa Bruno, Andrea Biondi, Sebastian Böttcher, Gergely Vértes, Richard Dobson, Amos Folarin, Yatharth Ranjan, Zulqarnain Rashid, Nikolay Manyakov, Aki Rintala, Inez Myin-Germeys, Sara Simblett, Til Wykes, Amanda Stoneman, Ann Little, Sarah Thorpe, Simon Lees, Andreas Schulze-Bonhage, Mark Richardson

**Affiliations:** 1 Institute of Psychiatry, Psychology & Neuroscience King's College London London United Kingdom; 2 Epilepsy Center, Department of Neurosurgery, Medical Center University of Freiburg Freiburg Germany; 3 Epilepsy Seizure Detection – Neurology UCB Pharma Brussels Belgium; 4 Feasibility Advanced Analytics, Clinical Insights and Experience, Janssen Research and Development Beerse Belgium; 5 Department of Neurosciences Center for Contextual Psychiatry KU Leuven Leuven Belgium; 6 Faculty of Social Services and Health Care LAB University of Applied Sciences Lahti Finland; 7 The RADAR-CNS patient advisory board King’s College London, UK London United Kingdom

**Keywords:** epilepsy, seizures, telemedicine, medical device, mobile phone

## Abstract

**Background:**

In recent years, a growing body of literature has highlighted the role of wearable and mobile remote measurement technology (RMT) applied to seizure detection in hospital settings, whereas more limited evidence has been produced in the community setting. In clinical practice, seizure assessment typically relies on self-report, which is known to be highly unreliable. Moreover, most people with epilepsy self-identify factors that lead to increased seizure likelihood, including mood, behavior, sleep pattern, and cognitive alterations, all of which are amenable to measurement via multiparametric RMT.

**Objective:**

The primary aim of this multicenter prospective cohort study is to assess the usability, feasibility, and acceptability of RMT in the community setting. In addition, this study aims to determine whether multiparametric RMT collected in populations with epilepsy can prospectively estimate variations in seizure occurrence and other outcomes, including seizure frequency, quality of life, and comorbidities.

**Methods:**

People with a diagnosis of pharmacoresistant epilepsy will be recruited in London, United Kingdom, and Freiburg, Germany. Participants will be asked to wear a wrist-worn device and download ad hoc apps developed on their smartphones. The apps will be used to collect data related to sleep, physical activity, stress, mood, social interaction, speech patterns, and cognitive function, both passively from existing smartphone sensors (passive remote measurement technology [pRMT]) and actively via questionnaires, tasks, and assessments (active remote measurement technology [aRMT]). Data will be collected continuously for 6 months and streamed to the Remote Assessment of Disease and Relapse-base (RADAR-base) server.

**Results:**

The RADAR Central Nervous System project received funding in 2015 from the Innovative Medicines Initiative 2 Joint Undertaking under Grant Agreement No. 115902. This Joint Undertaking receives support from the European Union’s Horizon 2020 research and innovation program and European Federation of Pharmaceutical Industries and Associations. Ethical approval was obtained in London from the Bromley Research Ethics Committee (research ethics committee reference: 19/LO/1884) in January 2020. The first participant was enrolled on September 30, 2020. Data will be collected until September 30, 2021. The results are expected to be published at the beginning of 2022.

**Conclusions:**

RADAR Epilepsy aims at developing a framework of continuous data collection intended to identify ictal and preictal states through the use of aRMT and pRMT in the real-life environment. The study was specifically designed to evaluate the clinical usefulness of the data collected via new technologies and compliance, technology acceptability, and usability for patients. These are key aspects to successful adoption and implementation of RMT as a new way to measure and manage long-term disorders.

**International Registered Report Identifier (IRRID):**

PRR1-10.2196/21840

## Introduction

### Background

The last decade has seen an explosion in the capability of monitoring individuals via sensors in smartphones or wearable devices, and the range of parameters that can be measured by such technologies will continue to grow [1]. Remote measurement technologies (RMTs) can unobtrusively measure human behavior and physiology and, combined with active measurement of daily experiences via smartphone apps, is an innovation that could be used to provide real-time information about the current clinical state of patients and information potentially predictive of future deterioration [1]. RMT appears to be particularly interesting for those chronic conditions whose course is dynamic and characterized by multiple variations in parameters that can be measured remotely, actively and/or passively [2,3]. Epilepsy is one of the most common neurological disorders, affecting approximately 0.6% of the population worldwide [4]. It is characterized by recurrent seizures that manifest with physiological and behavioral phenomena [5]. Seizures are mostly unprovoked and unpredictable, and about 1 in 3 of all people with epilepsy do not respond to any medication and continue to have uncontrolled seizures [6]. The personal, social, and economic costs of uncontrolled epilepsy are considerable: seizure recurrence and unpredictability worsen the quality of life (QoL) of people with epilepsy and their families [7], people with active epilepsy have 4 to 5 times higher standardized mortality ratios compared with seizure-free individuals [8-12], and the economic burden for the health system is significant across countries [13,14]. Despite new antiepileptic drugs coming to the market, there is limited evidence of a substantial difference to treatment-refractory patients [15], and overall, the mortality associated with epilepsy has not decreased in the last 50 years [16]. One reason for the failure of trials of new treatments is that it is difficult to collect objective data regarding relevant clinical outcomes (eg, number of seizures and response to treatment) and that subjective patient and family recall and reporting is of limited utility and largely unreliable [17]. Similarly, an objective and continuous collection of information on seizure occurrence, frequency, and distribution would guide a more tailored management and inform decisions on treatment optimization in the clinical setting. In the diagnostic setting, the gold standard for seizure detection is electroencephalography (EEG) in combination with video monitoring. This method has, by definition, a high sensitivity and a positive predictive value close to 100%. However, video EEG monitoring is not a practical procedure for long-term seizure tracking for different reasons: it cannot be implemented for more than a few days at a time, wearing EEG electrodes is uncomfortable and potentially stigmatizing for the patient, EEG requires considerable care by technicians to maintain a high signal quality, and analysis of videos and EEG signals are time consuming and costly [18].

The advent of small, wearable sensors embedded in devices and smartphones has progressively attracted attention in the epilepsy research field [19]. Wearable sensors can be worn comfortably for continuous monitoring of biosignals, and no input from the wearer is required to gather data (passive remote measurement technology [pRMT]). Different studies, mostly based on epilepsy monitoring units (EMUs), have demonstrated the possibility of capturing cardinal seizure manifestations using wearable sensors [20-26]. Movements presenting during seizures have been recorded with accelerometry and surface electromyography. Heart rate variations have been captured by wearable electrocardiogram (ECG) and photoplethysmography (PPG), and alteration of the autonomic nervous system has been recorded with ECG, PPG, and electrodermal activity (EDA) sensors, with different levels of signal and seizure detection accuracy [27].

However, although seizures are the hallmark of the condition, most people with epilepy also develop a range of comorbid conditions of variable severity, including mood disturbances and memory impairment, which are strong predictors of QoL [28]. Moreover, the majority of people with epilepsy self-identify seizure precipitants, usually identified as social and behavioral factors, such as stress, sleep deprivation, and fatigue, which lead to increased seizure likelihood [29]. Despite their clinical importance, there is limited evidence regarding the temporal trajectory of cognitive and psychiatric comorbidity of epilepsy, and insufficient studies have prospectively evaluated the correlation between seizure precipitants and seizure occurrence.

RMT provides tools to reliably measure mood states, cognition, thoughts, and behaviors in real time [30-32], with evidence highlighting the increased validity of this methodology in comparison with traditional retrospective reports [33]. Smartphone apps can be used to deliver validated questionnaires, cognitive games, speech tasks, or electronic diaries to provide fine-grained understanding of mood changes and stressors in the context of daily life (active remote measurement technology [aRMT]) [34]. Moreover, information gathered through smartphones, including GPS, communication logs, ambient noise, light levels, and screen interactions, has been successfully used to identify changes in sleep and activity patterns in psychiatric conditions [35,36] and represent an additional pRMT feature. Despite the growing body of research evidence, there has been limited assessment of the utility of multiparametric RMTs (aRMT and pRMT) in specific clinical populations, and a paucity of studies performed in the context of daily life have used wearables for people with epilepsy [25]. Remote Assessment of Disease and Relapse-Central Nervous System (RADAR-CNS) is an international research program, which has developed an open-source platform to support pRMT and aRMT data collection to assess and monitor 3 different clinical conditions, major depressive disorder [37], epilepsy, and multiple sclerosis, in the ambulatory setting.

This study reports the methods of a clinical study, RADAR Epilepsy, designed to test patients’ acceptability and the clinical utility of multiparametric RMT in a clinical population with epilepsy in the real-world environment.

### Study Development and Patients’ Involvement

Systematic reviews have examined the barriers and facilitators of RMT uptake [38]; surveys and service user focus groups have identified device preferences and the variables of interest to the specific patient group [39,40]. Alongside clinical experts, the RADAR-CNS consortium has a team dedicated to ensuring patient and public involvement at every stage of development. People with epilepsy took part in focus groups to gather their views on the outcomes of importance to patients and potential barriers and facilitators to engagement [40]. We have appointed several people with epilepsy, in collaboration with a national charity (Epilepsy Action), to join a patient advisory board to help steer the project. These members and people with epilepsy in a hospital-based setting were involved in user testing the wearable devices and smartphone apps, contributing to crucial design choices throughout [40]. All information sheets have been reviewed and approved by a local service user advisory board (Feasibility and Acceptability Support Team for Researchers) and patients seen in local epilepsy clinics to ensure their accessibility. We will continue to involve patients throughout this study and include them in the dissemination of research findings.

### Study Objectives

The main aims of the RADAR Epilepsy study are (1) to determine the usability and acceptability of RMT to provide real-time objective multidimensional indications of clinical state in individuals with epilepsy; (2) to assess the correspondence of patient-marked seizure events (via aRMT) with the data collected from the pRMT; and (3) To determine whether multiparametric RMT collected in populations with epilepsy can prospectively estimate variations in seizure occurrence and other outcomes, including seizure frequency, QoL, and comorbidities.

## Methods

### Study Design and Population

This study is a multicenter, prospective, observational, nonrandomized, and noninterventional cohort study in which 32 individuals with a diagnosis of epilepsy will be asked to download several RMT apps and use a wearable device for up to 6 months of follow-up. The inclusion and exclusion criteria are provided in Textboxes 1 and 2, respectively.

**Inclusion criteria.**

Inclusion criteria

- Able to give informed consent for participation
- Diagnosis of epilepsy
- Aged between 18 and 70 years
- Minimum average seizure frequency of 2 seizures per month
- Willing and able to complete self-reported assessments via smartphones
- Fluent in English (German in Freiburg) and able to read and write English (German in Freiburg)
- Willing to continuously wear the study wearable device
- Existing ownership of an Android smartphone (or willingness to use an Android smartphone, which will be provided as their only smartphone)
- Existing ownership or the possibility of connecting to a Wi-Fi connection on a daily basis

**Exclusion criteria.**

Exclusion criteria

- Established diagnosis of psychogenic nonepileptic attacks (dissociative seizures) as the only seizure type
- Frequent vigorous involuntary movements (eg, chorea and athetosis) or frequent parasomnias with major motor components (eg, sleep walking and night terrors)
- Inability to comply with the trial procedure, such as cognitive or behavioral problems
- Inability to give informed consent
- Unwillingness to use an Android smartphone

### Study Technology

The open-source RADAR-base platform [41] developed to support the RMT data collection is described elsewhere [42]. A number of variables will be collected from each patient through the RMT.

**Figure 1:**
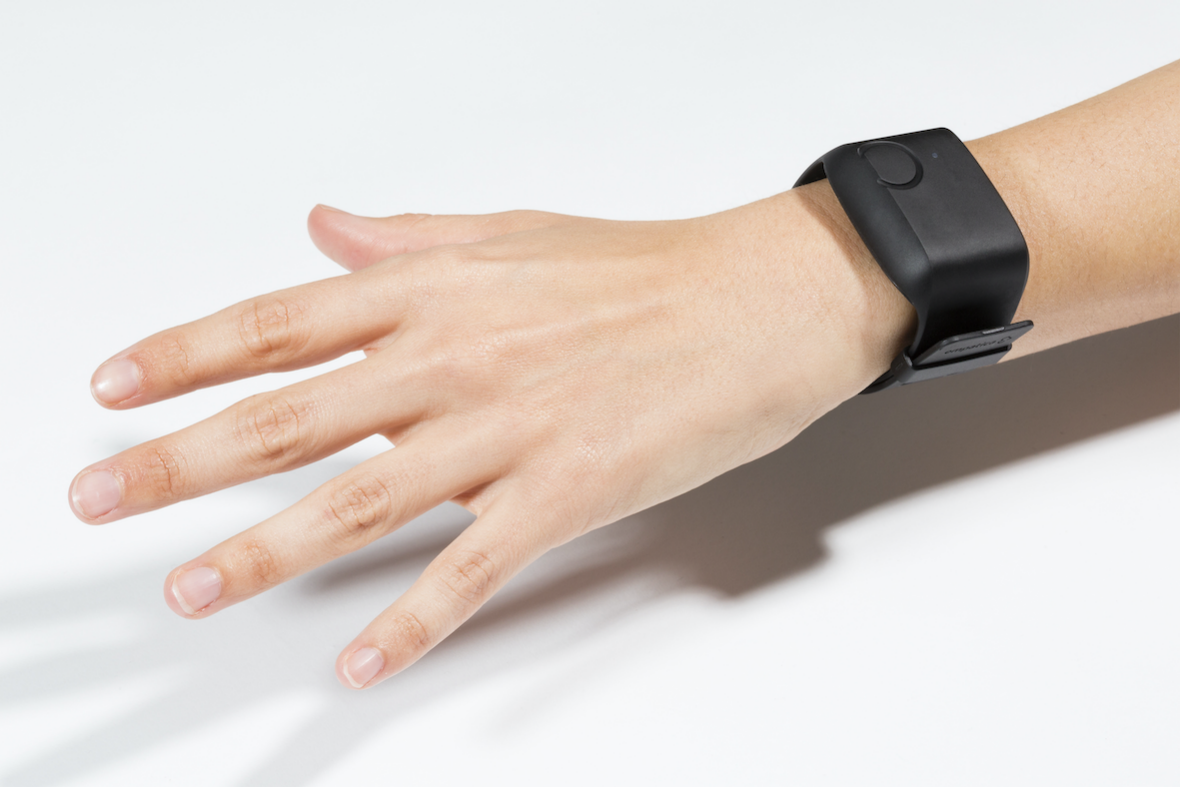
The Empatica E4 device.

pRMT includes the following:

1. Smartphone app: Participants will be assisted in downloading a purpose-built app that will run in the background, requiring no further input. Using sensors commonly present in all modern smartphones, the app will collect data on ambient noise, ambient light, GPS location, bluetooth connectivity, and battery life. GPS location data will be randomized (providing relative location data, not absolute coordinates).
2. Wearable device: Participants will be asked to wear the Empatica E4 device, a Conformitè Europëenne–marked wristband (Figure 1), for the entire duration of the study (6 months). The device will require a few hours of charging once daily, and participants will receive training and accessories to do so on a routine basis and a second E4 device to be worn during the charging procedure. The sensors included in this wearable device and the parameters measured are PPG and series of interbeat intervals derived from it, 3D acceleration, body temperature, and EDA.

A number of variables of interest to this study will be collected through a second purpose-built aRMT app, which will prompt the study participant to complete validated questionnaires and assessments according to the study schedule (Table 1):

1. Psychiatric comorbidities: Anxiety will be measured via the 7-item Generalized Anxiety Disorder Questionnaire [43]; depressive symptoms will be monitored via the 8-item Patient Health Questionnaire [44].
2. Seizure occurrence and precipitants: Each evening, participants will be prompted to complete a daily assessment to provide self-reported information about medication compliance, sleep quality, alcohol use, menstrual cycle, stress, mood, anxiety, and seizure occurrence. If a seizure is reported, the participant will be automatically directed to an ad hoc created seizure diary. Information on clinical symptoms and manifestations, time of the event, and possible precipitants will be collected through the diary.
3. Experience sampling method: Participants will be asked to complete a series of short electronic diary assessments, also known as the experience sampling method (ESM). ESM assesses experiences and behavior in the realm of daily life and will be administered to provide real-time self-reported data on mood, stress, cognition, activity, location, social interactions, physical state, and medication use. An ESM period will be initiated once every 6 weeks, during which participants will receive notifications to complete 9 questionnaires per day over a 6-day period (semirandom interval between 8:30 AM and 10 PM).
4. Speech task: On a random schedule, participants will be asked to hold down a button on their phone to start recording and will then be asked to say aloud, and in a quiet area, some excerpts from *The North Wind and the Sun*, which are shown to be phonetically balanced in different languages. In addition, participants will be asked to respond to the following question: *Can you describe something you are looking forward to this week?* These data will be recorded in a raw audio format, allowing speech recognition to extract content and automatic sentiment detection.
5. Cognitive function: Cognitive assessment will use the THINC-ITapp, which has been validated to screen for cognitive dysfunction [45] and examines executive functions, such as memory, attention, and concentration.

**Table 1:** Schedule of events for Remote Assessment of Disease and Relapse Epilepsy.

Events		Weeks (months)												
		0 (0)	2 (0)	4 (1)	6 (1)	8 (2)	10 (2)	12 (3)	14 (3)	16 (4)	18 (4)	20 (5)	22 (5)	24 (6)
Visit		1 (Baseline)	—^a^	2 (Follow-up)	—	—	—	3 (Follow-up)	—	—	—	—	—	4 (Study end)
**Assessment**														
	Study explanation	✓^b^	—	—	—	—	—	—	—	—	—	—	—	—
	Informed consent	✓	—	—	—	—	—	—	—	—	—	—	—	—
	Introductory training (60 min)	✓	—	—	—	—	—	—	—	—	—	—	—	—
	Sociodemographics	✓	—	—	—	—	—	—	—	—	—	—	—	—
	Medical history	✓	—	—	—	—	—	—	—	—	—	—	—	—
	Study debrief	—	—	—	—	—	—	—	—	—	—	—	—	✓
	Monitoring telephone call	—	✓	—	✓	✓	✓	—	✓	✓	✓	✓	✓	—
	Qualitative interview	—	—	✓	—	—	—	✓	—	—	—	—	—	✓
	TAM-FF^c^	—	—	✓	—	—	—	✓	—	—	—	—	—	✓
	PSSUQ^d^	—	—	✓	—	—	—	✓	—	—	—	—	—	✓
**Passive RMT ^e^**														
	Wearable sensors	—	—	—	—	—	—	Continuous	—	—	—	—	—	—
	Smartphone sensors	—	—	—	—	—	—	Continuous	—	—	—	—	—	—
**Active** **RMT**														
	Seizure diary and precipitants	—	—	—	—	—	—	Daily questionnaire	—	—	—	—	—	—
	ESM^f^ assessment	✓	—	—	✓	—	—	✓	—	—	✓	—	—	✓
	Cognition	✓	—	—	✓	—	—	✓	—	—	✓	—	—	✓
	Speech task	✓	✓	✓	✓	✓	✓	✓	✓	✓	✓	✓	✓	✓
	Mood PHQ8^g^ (A^h^)	✓	✓	✓	✓	✓	✓	✓	✓	✓	✓	✓	✓	✓
	Anxiety GAD7^i^ (A or W^j^)	✓	—	—	—	—	—	✓	—	—	—	—	—	✓
	Self-esteem RSES^k^ (A)	✓	—	—	—	—	—	✓	—	—	—	—	—	✓
	Quality-of-life WSAS^l^ (A or W)	✓	—	—	—	—	—	✓	—	—	—	—	—	✓
	BIPQ^m^ (A or W)	✓	—	—	—	—	—	✓	—	—	—	—	—	✓
	Change in medications (T^n^ or F2F^o^)	—	—	✓	—	—	—	✓	—	—	—	—	—	✓
	CSR^p^ (T or F2F)	—	—	✓	—	—	—	✓	—	—	—	—	—	✓
	LTE-Q^q^ (T or F2F)	—	—	✓	—	—	—	✓	—	—	—	—	—	✓

^a^—: Task or assessment not required.

^b^✓: Task or assessment required.

^c^TAM-FF: Technology Assessment Model Fast Form.

^d^PSSUQ: Post-Study System Usability Questionnaire.

^e^RMT: remote measurement technology.

^f^ESM: experience sampling method.

^g^PHQ8: 8-item Patient Health Questionnaire.

^h^A: assessed via app.

^i^GAD7: 7-item Generalized Anxiety Disorder questionnaire.

^j^W: web-based assessment.

^k^RSES: Rosenberg Self-esteem Scale.

^l^WSAS: Work and Social Adjustment Scale.

^m^BIPQ: Brief Illness Perceptions Questionnaire.

^n^T: telephone assessment.

^o^F2F: face to face assessment.

^p^CSRI: Client Service Receipt Inventory.

^q^LTE-Q: List of Threatening Experiences Questionnaire.

### Study Procedures

Participants will be recruited over 6 months from the Epilepsy Service and Clinical Neurophysiology Department at King’s College Hospital, London, and from the Epilepsy Center, Department of Neurosurgery, University of Freiburg, Germany.

Potentially eligible individuals will be identified among those attending a routine outpatient appointment or hospital video–EEG EMU admission or home video–EEG monitoring appointment at the participating sites. These individuals will be approached by a member of the on-site research team and given the study information sheet and consent form.

### Study Overview

The study schedule of events and assessments is detailed in Table 1.

After a minimum of 24 hours from the time they receive the study information sheet and consent form, the research team will contact the potential participant to assess their interest in taking part in the study. Following a positive response, a first visit will be scheduled for them at the research center to confirm eligibility, discuss the study, explain study procedures, answer any questions, and obtain written informed consent. Sociodemographic information, medical history, and baseline data will be collected (Table 1).

Participants will also receive a 60-min introductory training session on the use of the wearable device and will be assisted in downloading the apps. A leaflet summarizing key information will be provided. At the end of the training session, study participants will be asked to start wearing the device, and active and passive recording of data will begin. Participants will receive a follow-up call 2 weeks after the start of data recording to provide any additional support as required (Table 1).

After this, participants will attend a follow-up visit after 1 and 3 months (visits 2 and 3), where primary outcomes (as given in the *Study Outcomes* section) will be assessed. At visit 4 (study endpoint), participants will be invited to have a last face-to-face meeting to assess outcomes. A debrief session of 60 min will take place at visit 4, and acceptability and usability of the technology will be assessed with a qualitative interview and questionnaires (as given in the *Study Outcomes* section).

### Study Outcomes

The primary outcome of interest was participants’ acceptability and usability of the RADAR technology, including the wearable, the apps, and the assessment scheme. Acceptability and usability will be evaluated using the Post-Study System Usability Questionnaire (PSSUQ) [46]; the Technology Assessment Model Fast Form (TAM-FF) [47]; and ad hoc created qualitative interviews undertaken at 1, 3, and 6 months postenrollment. Participants who dropped out before the study endpoint will be contacted to complete an interview.

Secondary outcomes include (1) sensitivity and false alarm rate of the device, as compared with patient seizure diary, for different seizure types: generalized tonic-clonic seizures, focal seizures (FS) with motor features, FS with autonomic features, and FS with dyscognitive features and (2) identification of variables influencing the long-term trajectory of seizure frequency: pRMT features (measured through the wearable sensors and smartphone sensors), and aRMT assessments, including comorbidities, seizure precipitants, ESM, speech, and cognitive functions.

### Contextual Variables

Variation in self-esteem will be measured using the Rosenberg Self-Esteem Scale [48], a 10-item self-reported questionnaire. QoL or disability will be measured using the Work and Social Adjustment Scale [49], a 5-item assessment of perceived social and work-related functional impairment used widely across a range of mental and physical disorders. Illness perceptions will be assessed using the Brief Illness Perceptions Questionnaire [50], which provides an insight into participants’ views about their underlying conditions, including a measure of how well they see themselves coping with its symptoms and treatments.

We will seek to identify major changes in participants’ circumstances that may materially affect participants’ outcomes and may also impact the use of RMT. In particular, we will collect information about change in medications for epilepsy, health service utilization (the modified Client Service Receipt Inventory [CSRI]) [51], and change in life circumstances (List of Threatening Experiences Questionnaire [LTE-Q]) [52].

### Study Monitoring

In addition to evidence of the acceptability and usability of the RADAR technology, data obtained throughout the course of the study will be used to make iterative developments to the platform software and to the questionnaire schedule and to optimize the usability of the system. During the scheduled monitoring telephone calls, the correct use of device and apps will be assessed to identify whether a loss of data is because of potential technical or practical issues that can be resolved. Participants’ problems, concerns, and questions will also be discussed to facilitate engagement and to support adherence to the protocol. These contacts will be recorded as evidence of feasibility and acceptability outcomes. The research team will systematically review upcoming data and may decide to schedule additional monitoring telephone calls if some discrepancies were found during the data collection and streaming.

### Study Withdrawal

There may be several reasons for withdrawal from the study. Participants were free to withdraw from the study at any point without providing reasons. The research team may withdraw the participant in the event of intercurrent illness, adverse events, protocol violations, and administrative or other reasons. In the case of participant self-withdrawal, all attempts will be made to have a face-to-face appointment with the participant to establish the cause of withdrawal and to collect qualitative data regarding the experience of participation. All data, including those from study withdrawals (unless participants request for deletion of their data), will be included in the final analysis.

### Data Handling and Confidentiality

All digital and nondigital information related to study participants will be nonidentifiable, in accordance with the General Data Protection Regulation. Each participant will be assigned a sequential identification number, used to collect, store, and report participant information. Data acquired from the wearable devices and from the apps will be encrypted, anonymized, and uploaded automatically via Wi-Fi to secure servers and infrastructures (RADAR platform) managed by the research team.

### Statistical Analysis Plan

Descriptive statistics for demographics, attrition rate, and number of participants using remote assessment measurements will be estimated. The primary outcome measurement (PSSUQ and TAM-FF) will be analyzed using a mixed-effect model for repeated measures based on observed case data. The model will include the duration in the study and contextual variables (CSRI, LTE-Q, etc) as covariates. Using classification approaches, we will investigate whether any demographics and/or other numerical information might serve as a predictor for subjects dropout. Using mixed-effect models with participants as a random factor, we will estimate whether there is a univariate relationship between the amount of RMT usable data during the weeks before the outcome assessment and obtained scale measurements (PSSUQ and TAM-FF). Acoustic features such as pitch, jitter, shimmer, formants, and intensity of the voice will be extracted from the speech task and will be analyzed.

To assess seizure detection performance of a multiparametric wearable system, we will relate seizures detected by the wearable system to the information from the seizure diary, kept by each participant. We will estimate the sensitivity of seizure detection by the wearable system (ie, whether all seizures reported in the diary were detected by wearable system) and false alarm rate (ie, number of seizures detected by the wearable system but not reported in the diary per unit of time, eg, day).

We will develop an analysis strategy for managing multi-sensor data. To this end, we will investigate what type of features derived from the pRMT and aRMT correlate with seizure occurrence and frequency per unit of time (eg, day and week). Seizure occurrence and frequency will be derived from the multiparametric wearable system and from seizure diaries and analyzed separately. Data obtained from wearable biosensors will be used to derive information about participants’ activity during the day and information about sleep and mood. Aggregated features obtained from biosensors, smartphones, and cognitive tests and from ESM and their changes from baseline or previous time points (delta features) will be used for statistical analysis. First, we will use a univariate approach, where the number of seizures per unit of time will be modeled with each variable or feature as a fixed effect and participant as a random effect. Demographics and other baseline characteristics will be added to the model when necessary. Correction for multiple comparisons will be taken into account. Second, using aggregated data obtained through some time duration to predict the number of seizures per the same time duration, we will construct predictive models. Under the assumption that data are missing at random, multiple imputation will be applied for covariates, where missingness is not drastically high. Multivariate prediction models will be constructed, and variable selection, based on, for example, least absolute shrinkage and selection operator L1-regularization for linear models, or different feature selection algorithms will be applied. Model performance will be characterized through cross-validation, putting stress on the sensitivity and specificity of the predictive model.

### Qualitative Data Analysis

A thematic analysis will be performed on the interviews. Once transcribed, the topics of interest will be identified. A framework will be developed to guide indexing of the major themes and subthemes. All data extracts will be reviewed for coherence and further refinement of the framework. Once the themes and subthemes have been identified, a framework matrix will be constructed to create summaries of the data. There will be a further step of performing a secondary analysis to categorize and classify the extracted dimensions. A final written summary of explanations for these dimensions will be presented.

### Ethics Approval

RADAR Epilepsy will be conducted per the Declaration of Helsinki and Good Clinical Practice, adhering to the principles outlined in the National Health Service (NHS) *Research Governance Framework for Health and Social Care* (2nd edition). Ethical approval was obtained in London from the Bromley Research Ethics Committee (REC reference: 19/LO/1884) and in Freiburg from the Ethics Committee at the University of Freiburg. All staff working on the study have received training in study conduct, informed consent, and risk assessment. RMT data will be pseudonymized and stored in a research database in accordance with the General Data Protection Regulation. The informed consent process will ensure that participants understand the nature of the study and the data being collected. Interested individuals will be provided with the study materials, including information sheets and consent forms for review. If, after reading, they wish to participate, they will be invited to an enrollment session that will involve the collection of written consent before the administration of any study procedures. They will understand that their privacy is protected and that they can withdraw at any time without giving a reason and request to have all data collected from them deleted.

## Results

The RADAR-CNS project has received funding in 2015 from the Innovative Medicines Initiative 2 Joint Undertaking under Grant Agreement No. 115902. This Joint Undertaking receives support from the European Union’s Horizon 2020 research and innovation program and European Federation of Pharmaceutical Industries and Associations (EFPIA). Ethical approval was obtained in London from the Bromley Research Ethics Committee (REC reference: 19/LO/1884) in January 2020. The first study participant was enrolled on September 30, 2020. Data will be collected until September 30, 2021. The results of the study are expected to be published at the beginning of 2022.

## Discussion

### Principal Findings

Epilepsy is a complex neurological condition characterized by seizures and by multiple factors that have been demonstrated to affect seizure control. The development of automated seizure detection systems is important for many reasons. In clinical practice, a more objective and continuous collection of information on seizure occurrence, frequency, and distribution during the day and night would more consistently guide patient management and decision-making processes, including treatment. Moreover, objective measures are required to improve outcome assessments in clinical trials. Finally, and most importantly, people with epilepsy have largely expressed their interest in digital tools for seizure detection and a desire to use multimodal devices to supplement major unmet needs, such as improving safety and self-management and providing reassurance to self and others [39,40]. Acquiring objective evidence of accuracy and timing of seizure detection is very important in this context, as false alarms can increase distress and have a detrimental impact on technology acceptance and long-term use [39,40].

Although a growing body of literature has focused on mobile biosensors for seizure detection, the majority of these studies are performed in hospital environments; hence, data from ambulatory studies are required. Moreover, to meet the needs of people living with epilepsy and ensure tools that can significantly improve their QoL, it appears necessary to think beyond seizures.

In the general epilepsy population, psychiatric comorbidities are frequent, up to 90% identify the presence of at least one seizure precipitant and many report feelings preceding their seizures, including mood, behavior, and cognitive alterations (premonitory features) [53-55]. The presence of complex interactions between seizure precipitants and premonitory features [56,57] makes the assessment of their real impact on seizure risk variation difficult. In addition, studies performed so far are lacking methodological tools that allow an objective and adequate assessment of precipitants, including a measurement of their sudden variation. In fact, irrespective of their retrospective or prospective design, research studies have surveyed patients with the use of cross-sectional questionnaires and diaries, which are subject to recall bias and lack of time stamps. Another major methodological issue of the current literature data is related to the choice of arbitrary time windows between the assessment of a specific factor and the consequent occurrence of a seizure, usually using the data collected the day before to speculate about their influence on seizures occurring the following day. Prediction might be more robust over short time frames for some precipitants and premonitory features and over long time periods for others. Moreover, it is not clear whether a single report of a status (eg, stress or anxiety) reflects the average over the course of a day [58], and multiple collection points per day might be necessary to take into account symptom fluctuations and variations from day to day or moment to moment.

A complete assessment of seizure occurrence, precipitants, premonitory features, and their interactions cannot leave aside all the aforementioned considerations. RADAR Epilepsy aims to overcome the current methodological issues and to develop an ideal framework of continuous data collection intended to identify ictal and preictal states through the use of aRMT and pRMT. Context-sensitive fine-grained assessments with an excellent temporal resolution in the context of daily life are the hallmarks of RADAR Epilepsy.

### Conclusions

Currently, it is paramount to find innovative, safe, and reliable ways to monitor and manage patients with epilepsy at home. RMT may soon represent a new way to measure and manage long-term disorders in a real-life environment, and RADAR Epilepsy is a step forward to achieve this goal. Our future work will have the objective of specifically evaluating the clinical usefulness of the data collected via RMT and compliance, technology acceptability, and usability for patients. RADAR Epilepsy has been specifically designed to primarily evaluate these aspects and the potential challenges to the successful adoption and implementation of RMT in conventional health care systems.

## Appendix

Multimedia Appendix 1 Peer Review Report.

## Abbreviations

aRMT: active remote measurement technology
CSRI: Client Service Receipt Inventory
ECG: electrocardiogram
EDA: electrodermal activity
EEG: electroencephalography
EFPIA: European Federation of Pharmaceutical Industries and Associations
EMU: epilepsy monitoring units
ESM: experience sampling method
FS: focal seizures
LTE-Q: List of Threatening Experiences Questionnaire
NHS: National Health Service
NIHR: National Institute for Health Research
PPG: photoplethysmography
pRMT: passive remote measurement technology
PSSUQ: Post-Study System Usability Questionnaire
QoL: quality of life
RADAR Epilepsy: Remote Assessment of Disease and Relapse Epilepsy
RADAR-CNS: Remote Assessment of Disease and Relapse-Central Nervous System
RMT: remote measurement technology
TAM-FF: Technology Assessment Model Fast Form
