# Supplementary material for: Remote Assessment of Disease and Relapse in Epilepsy: Protocol for a Multicenter Prospective Cohort Study
Source: JMIR Res Protoc. 2020 Dec 16;9(12):e21840. doi: 10.2196/21840 (PMC7773514; doi:10.2196/21840)
Supplement: Multimedia Appendix 1 [file resprot_v9i12e21840_app1.pdf]

Proposal N° : 115902-2	Acronym : RADAR-CNS

Proposal Evaluation Form

innovative medicines initiative	**INNOVATIVE MEDICINES INITIATIVE**	**Evaluation Summary Report**<br>**Research and innovation actions**

Call: H2020-JTI-IMI2-2015-03-two-sta
Topic: IMI2-2015-03-01
Proposal Number 115902-2
Proposal Acronym RADAR-CNS
Proposal Title Remote Assessment of Disease and Relapse in Central Nervous System Disorders

Evaluation Summary Report

Evaluation Result

Total score: 13.00

Scoring

0 *The **proposal fails to address the criterion** or cannot be assessed due to missing or incomplete information.*
1 ***Poor**. The criterion is inadequately addressed, or there are serious inherent weaknesses.*
2 ***Fair**. The proposal broadly addresses the criterion, but there are significant weaknesses.*
3 ***Good**. The proposal addresses the criterion well, but a number of shortcomings are present.*
4 ***Very good**. The proposal addresses the criterion very well, but a small number of shortcomings are present*
5 ***Excellent.** The proposal successfully addresses all relevant aspects of the criterion. Any shortcomings are minor.*

Operational Capacity

❄ All the partners in this proposal possess the basic operational capacity to carry out the proposed work
⭘ The following partner(s) lack(s) the basic operational capacity to carry out the proposed work, for the reasons indicated. The proposal has been evaluated by disregarding the partner(s) concerned, the activities assigned to them, and the corresponding budget.

Criterion 1 – Excellence

Score: **4.50** (Threshold 3.00/5.00, Weight: 100.00%)

Note: The following aspects will be taken into account, to the extent that the proposed work corresponds to the topic description in the IMI2 annual work plan:

- **Clarity and pertinence of the objectives;**
- **Credibility of the proposed approach;**
- **Soundness of the concept, including trans-disciplinary considerations, where relevant;**
- **Extent that proposed work is ambitious, has innovation potential, and is beyond the state of the art;**
- **Mobilisation of the necessary expertise to achieve the objectives of the topic and to ensure engagement of all relevant key stakeholders.**

Comment

The objectives are clear, concise and very well aligned with the call text

The proposal clinical strategy has been well developed from the SP especially in MDD and MS.

Taking established sensor technology should facilitate a rapid start to the project, however, the consortium must remain flexible to technologies that emerge during the project lifetime.

The planned use of both general monitoring and comprehensive monitoring in a controlled environment is particularly welcomed.

The proposed concept appears to be sound and combines several disciplines. However, further information on the system design would have strengthened the proposal.

Proposal N° : 115902-2	Acronym : RADAR-CNS

The panel welcomed the consortium's ambition to include an end to end plan to translate RMT technology to daily healthcare practice. The panel also felt that the proposal to ensure the platform is scalable beyond the three selected conditions and to disease prevention in healthy populations shows good innovation potential.

The design of the epilepsy study would have benefited from a greater ambition.

As mentioned in the SP evaluation report: The proposal could have benefited from a more comprehensive survey of background information of existing findings and other research projects.

The consortium contains most of relevant expertise required, including several big, well connected clinical centres. The inclusion on expertise on behavioural change and of young researchers is especially commended.

The panel welcomes the strong regulatory, clinical development and healthcare pathways expertise which should ensure the results translate to daily medical care.

Expertise in statistics and big data management was not adequately described in the proposal, though the panel acknowledges the willingness of EFPIA to provide these resources as the project progresses, as clarified during the hearing.

The Scientific Advisory Board has very impressive clinical expertise but would also benefit from the inclusion of representatives from the technology industries.

Criterion 2 – Impact

Score: **4.50** (Threshold 3.00/5.00, Weight: 100.00%)

Note: The following aspects will be taken into account, to the extent to which the outputs of the project should contribute at the European and/or International level:

- **The expected impacts of the proposed approach listed in the IMI2 annual work plan under the relevant topic;**
- **Enhancing innovation capacity and integration of new knowledge;**
- **Strengthening the competitiveness and industrial leadership and/or addressing specific societal challenges;**
- **Improving European citizens' health and wellbeing and contribute to the IMI2 objectives[1] ;**
- **Any other environmental and socially important impacts;**
- **Effectiveness of the proposed measures to exploit and disseminate the project results (including management of IPR), to communicate the project, and to manage research data where relevant.**

[1] Article 2 of the Council Regulation (EU) No 557/2014 of 6 May 2014 establishing the Innovative Medicines Initiative 2 Joint Undertaking (O.J. L169 of 7.6.2014)

Comment

The proposal successfully addresses the expected impacts of the topic text.

The consortium plans to develop an open platform with the aim of stimulating further participation by organisations outside the consortium, which should increase the impact on innovation capacity. This could strengthen the competitiveness and industrial leadership of Europe, especially with regard to SMEs.

The panel appreciated the strong participation of patients which should improve European citizens' health and wellbeing by ensuring the translation of the results to daily medical practice, especially early intervention. The panel also felt the aim to explore the effects of different cultures and healthcare systems was a strength.

The dissemination plan was standard, especially for such an engaging field. To maximise the impact, the proposal would have benefited from making use of the networks and PR departments of the large companies to disseminate their results.
The panel was concerned that IP was not fully elaborated in the proposal, and noted that several proprietary software solutions are going to be used.

Criterion 3 – Quality and efficiency of the implementation

Score: **4.00** (Threshold 3.00/5.00, Weight: 100.00%)

Note: The following aspects will be taken into account, to the extent to which the outputs of the project should contribute at the

Proposal N° : 115902-2	Acronym : RADAR-CNS

European and/or International level:

- **Coherence and effectiveness of the project work plan, including appropriateness of the allocation of tasks and resources;**
- **Complementarity of the participants within the consortium (where relevant);**
- **Clearly defined contribution to the project plan of the industrial partners (where relevant);**
- **Appropriateness of the management structures and procedures, including risk and innovation management and sustainability plan.**

Comment

The panel felt that the work plan was coherent and that the resources were allocated appropriately. The decision to split some of the work-packages was welcomed, as was the organisation into clusters.

The proposal would benefit from a review of the number and timing of deliverables to ensure effective communication across the work packages, and allow for effective project and risk management. This is particularly important for such a large consortium.

The skills within the consortium are complementary and appropriate. It is also clear that good working relationships have developed between the industry and academic participants.

The contribution of the industrial partners is well defined in each work package, and the industry lead seems particularly well engaged. The panel also welcomed the inclusion of two large IT companies.

The academic/EFPIA co-leadership model is welcomed as appropriate, though the panel felt that the Management Team would benefit from better balance towards the academic and technology domains.

The panel noted that recruiting patients for the focus groups/pilots from already motivated patients may lead to a biased result.

The panel felt that the risk analysis did not capture all the critical risks for the project and felt that the risks around consortium size & internal communication, partner turnover, technical problems with platform design and operation should have been included. The proposal would also benefit from a contingency plan.

Exceptional funding of partners from non-EU/non-associated countries.

Applicants from countries and international organisations that do not automatically receive EU funding, or where there is no provision noted in the work plan for funding, may exceptionally be granted funding in a project, at the discretion of the IMI2 JU, if their participation is deemed essential for carrying out the project (e.g. outstanding expertise; access to unique know-how; access to research infrastructure; access to particular geographical environments; involving key partners in emerging markets; access to data). We believe the following applicant(s) who have requested EU funding meet this condition, for the reasons given

NWU is bringing the Purple Robot software which is a key part of the technology platform and also behavioural expertise. Therefore the participation of NWU is deemed essential to the RADAR-CNS project.
